# Effectiveness of a physical activity intervention on the overweight and obesity of Chilean schoolchildren

**DOI:** 10.1097/MD.0000000000030908

**Published:** 2022-09-30

**Authors:** Andrés Godoy-Cumillaf, Paola Fuentes-Merino, Frano Giakoni-Ramírez, Daniel Duclos-Bastías, Eugenio Merellano-Navarro

**Affiliations:** a Universidad Autónoma de Chile, Chile. Grupo de investigación en Educación Física, Salud y Calidad de Vida. Pedagogía en Educación Física, Temuco, Chile; b Faculty of Education and Social Sciences, Universidad Andres Bello, Las Condes, Santiago, Chile; c Escuela de Educación Física, Pontificia Universidad Católica de Valparaíso, Valparaíso, Chile; d Departament of Physical Activity Sciences, Faculty of Education Sciences, Universidad Católica del Maule, Talca, Chile.

**Keywords:** cardiorespiratory, flexibility, motor skills, muscular strength, speed-agility

## Abstract

Physical fitness is considered a measure that integrates most of the functions of the human organism that are involved in physical activity. Motor competence is important due to its observed implications in the physical, mental and social development of children and adolescents. These assessments require the existence of updated reference values that allow the categorization of individuals and groups according to levels of physical fitness and motor competence. This paper describes the study protocol to Effectiveness of a Physical Activity Intervention on the Overweight and Obesity of Chilean Schoolchildren, and in this way determine, through the calculation of a representative sample, reference values for physical fitness and motor competence in children aged 4 to 6 years in the region of La Araucanía, Chile.

**Methods:**

children in pre-basic education in transition grades I and II (4 and 5 years old), and those in the first year of basic education (6 years old) will be invited to participate. The sample will be probabilistic. The measures of a randomized controlled trial (registered in ClinicalTrial.gov NCT04194580) will be used.

**Conclusion:**

the lack of reference values for physical condition and motor competence for children between 6 and 4 years of age in the Araucanía region highlights the need to establish values, which will contribute to improving the health of children of the age group to be worked on.

## 1. Introduction

Physical fitness is considered a measure that integrates most of the functions of the human organism that are involved in physical activity; its main components are cardiorespiratory endurance, muscular strength, speed-agility and flexibility.^[1]^ Scientific evidence indicates that presenting adequate physical fitness values are associated with health benefits such as having a healthier cardiovascular profile and lower risk of developing cardiovascular diseases throughout life^[2]^ improvements in mental health,^[3]^ decreased metabolic risk^[4]^ and total adiposity.^[5]^ Considering the aforementioned, it is convenient to include tests to assess the level of physical fitness of children in the school setting.^[6]^ Regarding Chile, there is a lack of indicators on physical fitness level in children aged 12 years or younger.^[7,8]^

Motor competence is defined as the degree of performance in a variety of motor tasks that are performed with good control and coordination of movements^[9]^ and is important due to its observed implications in the physical, mental and social development of children and adolescents.^[10,11]^ It is composed of fine and gross motor skills, the former being important in the academic and social fields^[10]^ and the latter as a basis for future motor competence.^[12]^ If children do not master these skills, they are likely to have limited opportunities to participate successfully in different physical activities throughout life.^[13]^ Delays or deficits in motor competence can affect fitness levels of children and adults.^[14]^

When considering early prevention of risk factors for noncommunicable diseases such as obesity, diabetes, cardiovascular diseases, among others, the assessment of physical fitness levels and motor competence is necessary for decision making aimed at promoting healthy behavior.

These assessments require the existence of updated reference values that allow the categorization of individuals and groups according to levels of physical fitness and motor competence, in addition to being established by sex since from early ages there are significant differences.^[15–18]^

Internationally, reference values have been established for children aged 4 to 6 years, both for physical fitness^[6,19,20]^ and motor competence.^[14,21]^ In Chile, the first reference values for physical fitness and motor competence in children aged 4 to 6 years were published in 2020^[22]^ which, being a contribution, has the limitation that the sample of 700 students was not representative of the region of La Araucanía, which does not allow inferences to be made to all students of similar age in the region.

Thus, this proposal arises from the need to improve the existing reference values, for which methodological strategies will be used to eliminate the limitations. The information provided by this study will serve as support for educational and health establishments, sports centers and parents, for the formulation of strategies that favor the adequate development of the variables studied in children from 4 to 6 years of age.

## 2. Objective

Effectiveness of a Physical Activity Intervention on the Overweight and Obesity of Chilean Schoolchildren, and in this way determine, through the calculation of a representative sample, reference values for physical fitness and motor competence in children aged 4 to 6 years in the region of La Araucanía, Chile.

## 3. Methods

### 3.1. Design

Non-experimental study, descriptive level and cross-sectional design, under a quantitative research approach, which will use the measurements of a randomized controlled trial registered in ClinicalTrial.gov (NCT04194580), whose objective is to evaluate the efficacy of an activity intervention physical therapy to prevent and treat obesity and excess weight in children aged 4 to 6 years.

### 3.2. Recruitment

For recruitment, an informative letter will be sent to educational establishments in the communes that make up the Araucanía region. The researchers will provide information about the objectives and methods of the study to the principal, school board and physical education teachers at the schools that agree to participate. The Scientific Ethical Committee of the Universidad Autónoma de Chile approved the study protocol (N°CEC 31-22).

### 3.3. Participants

In each establishment, children in pre-basic education in transition grades I and II (4 and 5 years old), and those in the first year of basic education (6 years old) will be invited to participate. Parents will be invited to an informative meeting where the researchers will provide the objectives and procedures of the study. The signing of an informed consent form by the parents will be mandatory for the children to participate. Information on each child’s parameters will be sent to the educational establishments as well as to the parents.

### 3.4. Inclusion and exclusion criteria

Once the sample calculation is estimated, which is detailed below, the following will be considered as inclusion criteria for schoolchildren: being enrolled in a school or college in the Araucanía region, belonging to the urban area, regardless of their administrative dependence; being in transition level I, II or first grade in 2022 and belonging to the age range of the study having authorization from the parents or legal guardians for the student’s participation in the research and that the children agree to participate in the study. The exclusion criteria for schoolchildren are suffering from pathologies that contraindicate the physical tests or that require special attention.

### 3.5. Outcome measures

The evaluations will be carried out between September and December 2022. They will be carried out by previously trained evaluators to ensure standardization.

Physical Condition: musculoskeletal capacity will be measured in the following criteria: upper body strength, through manual grip dynamometry; lower limb strength, through the standing long jump; speed-agility, through the 4 × 10 m test; cardiorespiratory capacity, will be measured through the Course-Navette test. These tests are part of the FITness in PREschoolers battery.^[23,24]^

Motor competence: will be measured through the Movement Assessment Battery for Children, in its second edition validated in Spanish,^[25]^ which has proven to be an instrument with adequate psychometric reliability properties (Cronbach’s α > 0.60; κ = 1; ICC = 0.85–0.99).^[26]^ This tool was developed for use in clinical and educational settings. The version with age range 4 to 6 years will be used, with the component’s manual dexterity, aiming and catching; balance (static and dynamic).

Anthropometric variables: weight will be obtained with a scale (Tanita MC-780U, Japan); height with a stadiometer (Seca 220, Germany); waist circumference, with a tape measure (Rosscraft, Canada); hip circumference, with a tape measure (Rosscraft, Canada). All variables will be measured twice, according to the protocol proposed by the International Society for Advances in Kineanthropometry.^[27]^ The average will be used for statistical analyses.

Body composition: Body mass index will be obtained through weight and height, as proposed by the World Health Organization.^[28]^ Waist-to-height and waist-to-hip ratios will be calculated to determine the level of central adiposity. Percentage body fat and fat-free mass will be measured using a bioimpedance analysis system (Tanita MC-780U, Japan).^[29]^

## 4. Data analysis and management

### 4.1. Sample

Since the study will evaluate a representative sample of the Araucanía region, the sample size will be calculated according to the following criteria: total enrollment of students in transition I, II, and first grade in 2021; confidence level of 95%; margin of error of 5%; response rate of 80%. A sample 20% higher than the minimum will be established.

### 4.2. Statistical analysis

The quantitative information collected will be tabulated in a database and analyzed using IBM SPSS Statistics 26. Descriptive values of frequencies, means and medians will be obtained according to the characteristics of the variables (continuous/discrete). The normality of the variables will be checked by means of graphs and the Kolmogorov-Smirnov test. Student *t* test or the Mann–Whitney *U* test (if the distribution is not normal) will be used to analyze differences between groups. In analyses involving more than 2 groups, analysis of variance or Kruskal-Wallis (if the data distribution is not normal) will be applied. Differences will be accepted considering a *P* value <.05. When statistically significant differences are found, the effect size will be calculated. Percentile values will be calculated using the least-mean-square algorithm method.^[30]^

## 5. Discussion

Because physical fitness and motor competence assessments are necessary to make decisions to prevent or treat risk factors for various noncommunicable diseases from an early age. This article provides the rationale and methods of a study aimed at determining reference values for physical fitness and motor competence in children aged 4 to 6 years in the Araucanía region, Chile. Although there is previous evidence that proposed reference tables, the limitations do not allow them to be used in all children of similar age in the region. This situation supports the need for additional evidence on the subject of the study.

Some of the limitations of this study are its cross-sectional nature, which in the absence of other variables does not allow for a better understanding of the phenomenon and the establishment of cause-and-effect relationships. The strength of this research is that it works with a population that has not been studied much, so the results found will help physical education and health professionals to identify those with low values of physical fitness and motor competence, which will allow establishing goals that will help improve their health. It will also contribute to the process of early talent detection, in those cases where high values are found in skills considered important for sport.

In conclusion, the lack of reference values for physical condition and motor competence for children between 6 and 4 years of age in the Araucanía region highlights the need to establish values, which will contribute to improving the health of children of the age group to be worked on.

## Author contributions

AGC designed the study. AGC is the principal investigator. PFM is the co-investigator. AGC will coordinate the study. AGC, PFM, FGR, DDB and EMN will carry out the study. EMN will provide statistical and epidemiological support. AGC and PFM will write the article with the support of FGR, DDB and EMN. AGC obtained the financing, with the help of PFM. All authors established the methods and questionnaires, provided comments on the drafts, read and approved the final version.

**Conceptualization:** Andrés Godoy-Cumillaf.

**Data curation:** Paola Fuentes-Merino.

**Formal analysis:** Paola Fuentes-Merino, Eugenio Merellano-Navarro.

**Funding acquisition:** Andrés Godoy-Cumillaf.

**Investigation:** Andrés Godoy-Cumillaf, Eugenio Merellano-Navarro.

**Methodology:** Paola Fuentes-Merino, Frano Giakoni-Ramírez.

**Project administration:** Andrés Godoy-Cumillaf, Daniel Duclos-Bastías.

**Software:** Frano Giakoni-Ramírez, Daniel Duclos-Bastías.

**Supervision:** Frano Giakoni-Ramírez.

**Validation:** Daniel Duclos-Bastías, Eugenio Merellano-Navarro.

**Writing – original draft:** Andrés Godoy-Cumillaf, Paola Fuentes-Merino.

**Writing – review & editing:** Frano Giakoni-Ramírez, Daniel Duclos-Bastías, Eugenio Merellano-Navarro.
